# Impact of Ectopic Pregnancy on the Outcomes of the Subsequent Pregnancy: A Systematic Review and Meta-Analysis

**DOI:** 10.3390/jcm14124112

**Published:** 2025-06-10

**Authors:** Dimitrios Papageorgiou, Ioakeim Sapantzoglou, Eleftherios Zachariou, Panagiotis Antsaklis, Georgios Daskalakis, Vasilios Pergialiotis

**Affiliations:** 1 Department of Gynecology, Athens Naval and Veterans Hospital, 11521 Athens, Greece; 2 1st Department of Obstetrics and Gynecology, National and Kapodistrian University of Athens, 11527 Athens, Greece; 3 1st Gynecology Department, Metropolitan General Hospital, 15562 Athens, Greece

**Keywords:** ectopic pregnancy, tubal pregnancy, preterm delivery, emergency cesarean section, gestational hypertension, placental abruption, low birth weight, salpingectomy, salpingostomy, methotrexate

## Abstract

**Background/Objectives:** Although ectopic pregnancy has been extensively studied in terms of epidemiology, associated risk factors, diagnostic approaches, and treatment modalities, the data regarding its impact on the development of adverse outcomes in subsequent pregnancy remain scarce and conflicting. We aim to evaluate the adverse perinatal outcomes of women with a history of ectopic pregnancy **Methods**: We used the Medline (1966–2024), Scopus (2004–2024), Clinicaltrials.gov (2008–2024), EMBASE (1980–2024), Cochrane Central Register of Controlled Trials CENTRAL (1999–2024), and Google Scholar (2004–2024) databases in our primary search. All studies that evaluated the impact of prior of ectopic pregnancy on the perinatal outcomes of the subsequent pregnancy and reported rates of adverse perinatal outcomes were considered eligible for inclusion. Twelve peer-reviewed papers were considered for inclusion in our study. We enrolled a total of 2,162,731 women. Of those, 23,823 (1.1%) had a history of prior ectopic pregnancy. A total of 4 out of 12 studies provided the necessary data to be included in the metanalysis. **Results**: Women with a history of treated ectopic pregnancy, either medically or surgically, demonstrated increased risk of developing placental abruption, hypertensive disorders of pregnancy, and preterm birth. History of ectopic pregnancy was also positively associated with low birth weight, subsequent ectopic pregnancy, and increased risk of a subsequent emergency cesarean section. **Conclusions**: The meta-analysis reveals evidence that ectopic pregnancy is positively associated with adverse perinatal outcomes in subsequent pregnancy. Our findings should be considered preliminary and serve as a basis for future research as the retrieved data are scarce and cannot be deemed sufficient.

## 1. Introduction

Ectopic pregnancy (EP) is a significant medical entity during the reproductive period, characterized by the implantation of the embryo outside the endometrial cavity, remaining one of the main causes of maternal morbidity and mortality worldwide. Although ectopic pregnancies account for approximately 2% of pregnancies [1], the incidence of ectopic pregnancy among women presenting to the emergency department with first-trimester bleeding and/or pain ranges from 6% to 16%. The majority of ectopic pregnancies occur as tubal pregnancies, which represent 95% of all cases, while ovarian and abdominal ectopic pregnancies remain rare. The management of ectopic pregnancy requires individualized care because its location determines both frequency and treatment protocols [2,3,4,5,6,7,8].

Although EP has been extensively studied in terms of epidemiology, associated risk factors, diagnostic approaches, and treatment modalities, the data regarding its impact on the development of adverse outcomes in the subsequent pregnancy remain scarce and conflicting [1,4,5]. The purpose of this systematic literature review and meta-analysis is to collectively assess obstetric data and adverse pregnancy outcomes from the subsequent pregnancies of women who have received treatment for ectopic pregnancy, either medically or surgically, and to potentially correlate those adversities with the presence of a previous ectopic pregnancy.

## 2. Materials and Methods

The present meta-analysis was conducted according to the Preferred Reporting Items for Systematic Reviews and Meta-Analyses (PRISMA) guidelines [9]. This study was based on data that had already been published in the international literature. Patient consent and institutional review board approval were not required in this type of study. This study protocol was published in PROSPERO before the conduct of this meta-analysis (registration number: CRD420251035191).

### 2.1. Eligibility Criteria, Information Sources, Search Strategy

Observational studies, both prospective and retrospective, as well as randomized trials that examined the effect of a prior ectopic pregnancy on the outcomes of the subsequent pregnancy (irrespective of the treatment modality used, medical or surgical), were considered eligible for inclusion. The treatment approach that was used to treat the prior ectopic pregnancy varied among the studies included, as indicated in the discussion section of the present article. Case reports, experimental studies, and conference proceedings were excluded from the present meta-analysis.

**Figure 1:**
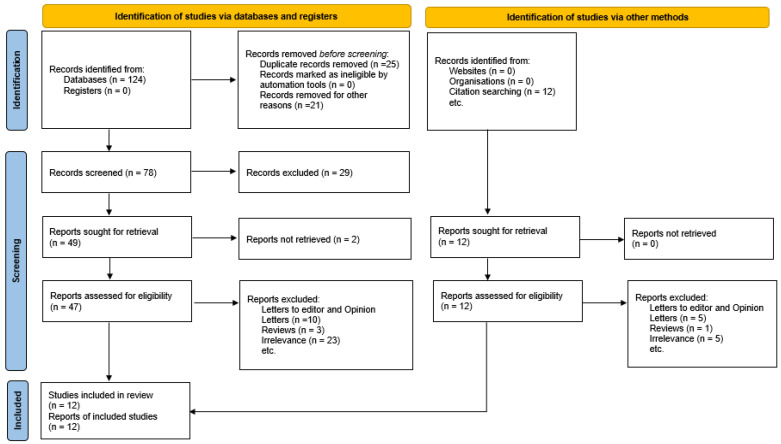
Flowchart of search strategy.

We used the Medline (1966–2024), Scopus (2004–2024), Clinicaltrials.gov (2008–2024), EMBASE (1980–2024), Cochrane Central Register of Controlled Trials CENTRAL (1999–2024), and Google Scholar (2004–2024) databases in our primary search along with the reference lists of electronically retrieved full-text papers. The date of our last search was set on 31 December 2024. Our search strategy included the text words “ectopic pregnancy; tubal pregnancy; CSP; interstitial pregnancy; subsequent pregnancy; adverse pregnancy outcomes; preterm delivery; preterm labor; placental abruption; hypertensive disorders of pregnancy; Preeclampsia; low birth weight; SGA; FGR; IUGR” and is briefly presented in Figure 1. The search identified 136 potentially relevant studies, but 124 were excluded because they were non-relevant articles or case reports and, in total, only 12 peer-reviewed papers were considered for inclusion in the current systematic review. Of those 12 studies, only 4 provided the necessary data and were eventually included in the meta-analysis.

### 2.2. Study Selection

Following deduplication, the titles and abstracts of all electronic articles were screened by three authors (D.P., I.S. and E.Z.) to assess their eligibility. The decision for inclusion of studies in the present systematic review and meta-analysis was taken after retrieving and reviewing the full version of articles that were considered potentially eligible. All studies that evaluated the impact of prior ectopic pregnancy on the perinatal outcomes of the subsequent pregnancy and reported rates of adverse perinatal outcomes were considered eligible for inclusion. Discrepancies that arose concerning the eligibility of retrieved studies were resolved by consensus from all authors.

### 2.3. Data Extraction

Outcome measures were predefined during the design of the present systematic review. Data extraction was performed using a modified data form that was based on Cochrane`s data collection form for intervention reviews for RCTs and non-RCTs. We predetermined as primary outcomes differences in the odds of placental abruption, preterm birth, hypertensive disorders of pregnancy, low birth weight, emergency caesarean section, and recurrent ectopic pregnancy.

### 2.4. Data Synthesis

The statistical meta-analysis was conducted with RStudio using the meta function (RStudio Team (2015) RStudio: Integrated Development for R. RStudio, Inc., Boston, MA, USA, URL http://www.rstudio.com/). Statistical heterogeneity was not taken into account when evaluating the suitable model (fixed effects or random effects) for statistical analysis, as the significant methodological heterogeneity (Table 1) precluded the assumption of comparable effect sizes among the studies included in the meta-analysis. Confidence intervals were established at 95%. We computed odds ratios (ORs) and 95% confidence intervals (CIs) using the Hartung–Knapp–Sidik–Jonkman method rather than the conventional DerSimonian–Laird random effects model analysis. The choice to conduct this analysis was made after considering recent findings that endorse its advantages over the Dersimonian–Laird model in comparing research with varying sample sizes and between-study variability.

The probable existence of small-study effects was intended to be assessed using Rücker’s Limit Meta-Analysis, while the likelihood of p-hacking was to be examined using the findings of the p-curve analysis. None of these analyses yielded solid results due to an insufficient number of trials. Prediction intervals (PIs) were computed by utilizing the meta function in RStudio to assess the anticipated effect expected in forthcoming investigations within the topic. The calculation of prediction intervals accounts for inter-study variability in the results and articulates the inherent heterogeneity on the same scale as the assessed outcome.

To assess the information size, we conducted trial sequential analysis (TSA), which allows for the examination of type I error in the aggregated results of meta-analyses conducted for the predefined primary outcomes in this meta-analysis. A minimum of three studies was deemed necessary to conduct the analysis. Repeated significance testing elevates the likelihood of type I error in meta-analyses, whereas TSA can recalibrate the intended significance level through the O’Brien–Fleming alpha-spending function. Consequently, during TSA, sequential interim analyses are conducted to evaluate the influence of each study on the overall results of the meta-analysis. The threshold for type I mistakes was established at 5%, while that for type II errors was set at 20%. The TSA analysis utilized TSA v. 0.9.5.10 Beta software (http://www.ctu.dk/tsa/, accessed on 3 February 2025).

### 2.5. Assessment of Risk of Bias

**Figure 2:**
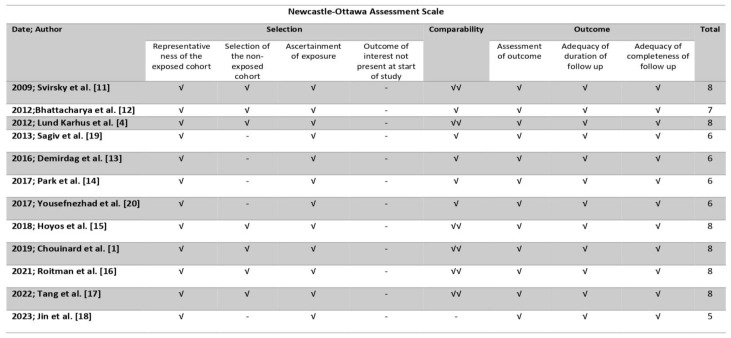
Newcastle–Ottawa Scale (NOS) quality assessment of the included studies. √: The selected quality item was evaluated and found present in the study (selection and outcome categories). √: The study groups were controlled for one important factor (comparability category). √√: The study groups were controlled for two important factors (comparability category) [1,4,11,12,13,14,15,16,17,18,19,20].

The methodological quality of all the included studies was evaluated using the Newcastle–Ottawa scale (NOS) tool, which is a widely utilized instrument for evaluating the methodological quality of non-randomized studies that are incorporated in systematic reviews and/or meta-analyses [10]. The tool assesses each study based on eight criteria, which are divided into three categories: the selection of study groups, the comparability of the groups, and the determination of either the exposure or outcome of interest for case–control or cohort studies, respectively. Stars were assigned to each quality item as a means of providing a rapid visual evaluation. Stars were allocated in a manner that granted the highest-caliber studies a maximum of nine stars. The tool was implemented by two authors independently, and any discrepancies were resolved through discussion with a third author. Overall, the risk of bias was assessed to be good, fair, or poor (Figure 2).

**Table 1:** Methodological characteristics of included studies.

Year	Author	Type of Study	Inclusion Criteria	Exclusion Criteria	Sample Size	Outcome Investigated
2009	Svirsky et al. [11]	Register-based retrospective cohort (2000–2006)	Women receiving MTX treatment for prior EP. Available subsequent pregnancy outcome information.	Missing data.	125	Duration of the pregnancy. Rate of late induced abortion. Rate of early and late missed abortions. U/S detection of abnormalities. Neonatal weight and overall condition.
2012	Bhattacharya et al. [12]	Register-based retrospective cohort (1981–2000)	Women with an EP in their first pregnancy (exposed group). Women who had a prior live birth (group A). Women who had a prior miscarriage (group B). Women who had a prior abortion (group C). Available subsequent pregnancy outcome information.	Missing data.	Exposed group: 2969 Group A: 667,299 Group B: 39705 Group C: 78,697	Rate of clinical pregnancy. Rate of live birth. Rate of EP. Rate of miscarriage. Rate of termination. Rate of stillbirth.
2013	Lund Karhus et al. [4]	Historical controlled cohort study (1977–2009)	Women with an EP in their first pregnancy (exposed group). Women who had a prior live birth (group A). Women who had a prior miscarriage (group B). Women who had a prior abortion (group C). Available subsequent pregnancy outcome information.	Missing data.	Exposed group: 2917 Group A: 2917 Group B: 2917 Group C: 2917.	Rate of clinical pregnancy. Rate of live birth. Rate of EP. Rate of miscarriage. Rate of termination. Rate of stillbirth.
2013	Sagiv et al. [19]	Case series (1997–2007)	Women with an interstitial EP treated by MTX, laparoscopy or laparotomy. Available subsequent pregnancy outcome information.	Women >40 years old. Women with bilateral salpingectomy or bilateral tubal ligation. Missing data.	14.	Fertility outcome.
2016	Demirdag et al. [13]	Register-based retrospective cohort (2007–2011)	Women with a tubal EP treated by MTX, surgery or expectantly. Available subsequent pregnancy outcome information.	Missing data.	112.	Rates of IUP. Rates of EP.
2017	Park et al. [14]	Retrospective Cohort (2010–2016)	Women with a tubal EP treated by MTX, surgery, or expectantly. Available subsequent pregnancy outcome information.	Missing data.	147.	Rate of clinical pregnancy. Rate of IU viable pregnancy. Rate of IU non-viable pregnancy. Rate of EP.
2017	Yousefnezhad et al. [20]	Cross-sectional study (2009–2013)	Women with an EP treated by MTX or surgery. Available subsequent pregnancy outcome information.	Missing data.	114.	Fertility rate. Rate of term and preterm pregnancies. Rate of abortions. Rate of EP.
2018	Hoyos et al. [15]	Retrospective cohort (2000–2013)	Women with a prior interstitial ectopic pregnancy that underwent cornual wedge resection. Available subsequent pregnancy outcome information.	Missing data.	19.	Rate of live birth. Rate of EP. Rate of abortion. Rate of adverse pregnancy outcomes.
2019	Chouinard et al. [1]	Population-based longitudinal cohort study (1989–2013)	Women with an EP in their first Pregnancy treated by surgery (exposed group). Women who had a prior live birth (unexposed group). Available subsequent pregnancy outcome information.	Missing data.	Exposed group: 15,823. Unexposed group: 110,1748.	Rate of live birth. Rate of EP. Rate of adverse pregnancy outcomes.
2021	Roitman et al. [16]	Retrospective cohort (1991–2004)	Women with an EP in their first pregnancy treated by surgery (group A). Pregnancy treated medically (group B). Women who had a prior live birth (unexposed group). Available subsequent pregnancy outcome information.	Multifetal pregnancies. Fetuses with congenital anomalies. Fetuses with chromosomal abnormalities. Missing data.	Group A: 1059. Group B: 365. Unexposed group: 242,258.	Rate of recurrent pregnancy loss. Rate of adverse pregnancy outcomes.
2022	Tang et al. [17]	Retrospective case–control (2014–2019)	Women with a prior interstitial ectopic pregnancy that underwent cornual wedge resection (exposed group). Women who had a prior live birth (unexposed group). All participants received frozen embryo transfer cycles of IVF/ICSI. Available subsequent pregnancy outcome information.	Women with uterine abnormities. Women with prior uterine surgery. Women with a personal history of diabetes, hypertension, or thyroid disorders. Missing data.	Exposed group: 75. Unexposed group: 375.	Rate of clinical pregnancy. Rate of live birth. Rate of EP. Rate of miscarriage. Rate of adverse pregnancy outcomes.
2023	Jin et al. [18]	Retrospective cohort (2009–2018)	Women with a prior treated CSP. Available subsequent pregnancy outcome information.	Missing data.	51	Subsequent fertility. Adverse pregnancy outcomes.

## 3. Results

### 3.1. Study Selection and Characteristics

Our search identified 136 potentially relevant studies, but 124 were excluded because they were non-relevant articles or case reports and, in total, only 12 peer-reviewed papers were considered for inclusion in the current systematic review, while 4 of them provided the necessary data to be included in the meta-analysis. Nine retrospective cohort studies were included in the present systematic review, with one of them being historically controlled and three of them registered based [4,11,12,13,14,15,16,17,18]. Additionally, one case series, one cross-sectional study, and one population based longitudinal cohort were included [1,19,20]. The studies enrolled a total of 2,162,623 women. Of those, 23,790 (1.1%) had a case of prior ectopic pregnancy. The methodological characteristics of the included studies are summarized in Table 1. The adverse perinatal outcomes under investigation in the subsequent pregnancy were the development of preterm birth prior to 37 weeks, placental abruption, emergency CS for fetal distress, hypertensive disorders of pregnancy (pregnancy induced hypertension, preeclampsia or HELLP), low birth weight, and the diagnosis of recurrent ectopic pregnancy. The diagnostic criteria of hypertensive disorders of pregnancy and preeclampsia were not defined in any of the studies that were included in the meta-analysis. For the outcome of low birth weight, only one of the three included studies defined it as birth weight of less than 2500 g.

### 3.2. Synthesis of Results

**Figure 3:**
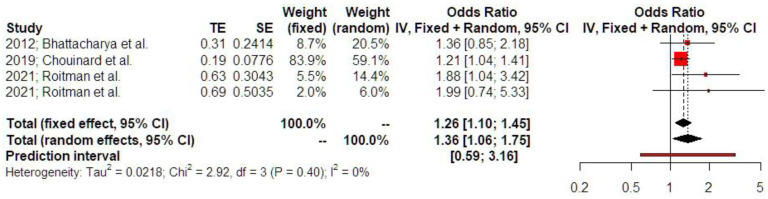
Odds ratio of placental abruption. The vertical line represents the “no difference” point between the two groups. Red squares represent the odds ratios of the included studies; horizontal black lines indicate the 95% confidence intervals of the included studies; diamond represents pooled odds ratios obtained from the meta-analysis outcomes, together with a 95% confidence interval for all studies; the horizontal red line represents prediction intervals. The weight of the included research is illustrated separately for fixed and random effects models. No statistical heterogeneity is noted for placental abruption (I^2^ = 0%) [1,12,16].

Women with a history of treated ectopic pregnancy, either medically or surgically, demonstrated increased risk of developing placental abruption (OR 1.36 [1.06; 1.75], findings from three studies). Prediction intervals indicated that the sample size that was used did not suffice to support these findings in future studies. The level of statistical heterogeneity was, however, particularly low (I-square test = 0%), which led to comparable effect estimates with the fixed effect model (OR 1.26, [1.10; 1.45]) (Figure 3).

**Figure 4:**
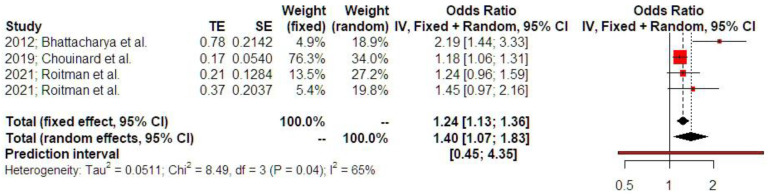
Odds ratio of hypertensive disorders of pregnancy. The vertical line represents the “no difference” point between the two groups. Red squares represent the odds ratios of the included studies; horizontal black lines indicate the 95% confidence intervals of the included studies; diamond represents pooled odds ratios obtained from the meta-analysis outcomes, together with a 95% confidence interval for all studies; the horizontal red line represents prediction intervals. The weight of the included research is illustrated separately for fixed and random effects models. Substantial statistical heterogeneity is noted for hypertensive disorders of pregnancy (I^2^ = 65%) [1,12,16].

The history of ectopic pregnancy was positively associated with subsequent hypertensive disorders of pregnancy (OR 1.40 [1.07; 1.83], findings from three studies). Prediction intervals indicated that the sample size that was used did not suffice to support these findings in future studies (Figure 4).

**Figure 5:**
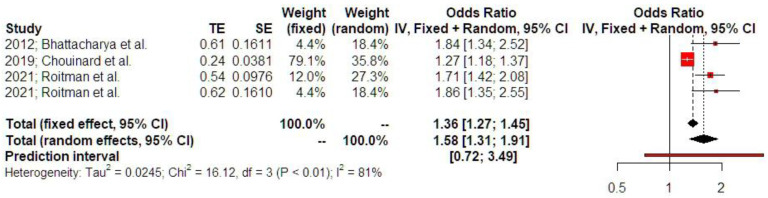
Odds ratio of preterm birth. Forest plot analysis: The vertical line represents the “no difference” point between the two groups. Red squares represent the odds ratios of the included studies; horizontal black lines indicate the 95% confidence intervals of the included studies; diamond represents pooled odds ratios obtained from the meta-analysis outcomes, together with a 95% confidence interval for all studies; the horizontal red line represents prediction intervals. The weight of the included research is illustrated separately for fixed and random effects models. High statistical heterogeneity is noted for preterm birth (I^2^ = 81%) [1,12,16].

A prior history of ectopic pregnancy was associated with increased odds of developing preterm birth <37 weeks (OR 1.58 [1.31; 1.91], findings from three studies). Prediction intervals indicated that the sample size that was used did not suffice to support these findings in future studies (Figure 5). P-curve analysis indicated the presence of evidential value and absence of p-hacking (Supplementary File S1).

**Figure 6:**
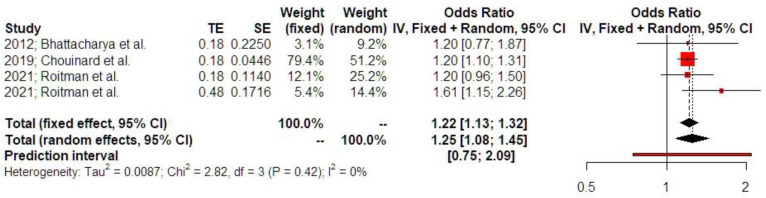
Odds ratio of low birth weight. Forest plot analysis: The vertical line represents the “no difference” point between the two groups. Red squares represent the odds ratios of the included studies; horizontal black lines indicate the 95% confidence intervals of the included studies; diamond represents pooled odds ratios obtained from the meta-analysis outcomes, together with a 95% confidence interval for all studies; the horizontal red line represents prediction intervals. The weight of the included research is illustrated separately for fixed and random effects models. No statistical heterogeneity is noted for low birth weight (I^2^ = 0%) [1,12,16].

A history of ectopic surgery was positively associated with low birth weight (OR 1.25 [1.08; 1.45], findings from three studies). Prediction intervals indicated that the sample size that was used did not suffice to support these findings in future studies. The level of statistical heterogeneity was, however, particularly low (I-square test = 0%), which led to comparable effect estimates with the fixed effect model (OR 1.22, [1.13; 1.32]) (Figure 6).

**Figure 7:**
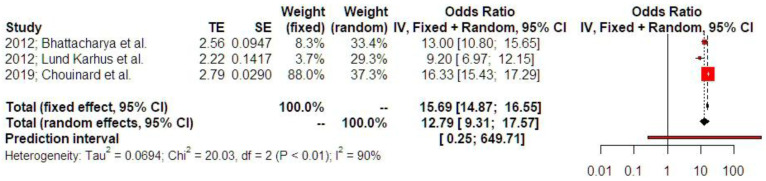
Odds ratio of subsequent ectopic pregnancy. The vertical line represents the “no difference” point between the two groups. Red squares represent the odds ratios of the included studies; horizontal black lines indicate the 95% confidence intervals of the included studies; diamond represents pooled odds ratios obtained from the meta-analysis outcomes, together with a 95% confidence interval for all studies; the horizontal red line represents prediction intervals. The weight of the included research is illustrated separately for fixed and random effects models. The weight of included studies is depicted for fixed and random effects model separately. High statistical heterogeneity is noted for subsequent ectopic pregnancy (I^2^ = 90%) [1,4,12].

Women with a history of treated ectopic surgery demonstrated an increased risk of subsequent ectopic pregnancy (OR 12.79 [9.31; 17.57], findings from three studies). Prediction intervals indicated that the sample size that was used did not suffice to support these findings in future studies (Figure 7). P-curve analysis indicated the presence of evidential value and absence of p-hacking (Supplementary File S1).

**Figure 8:**
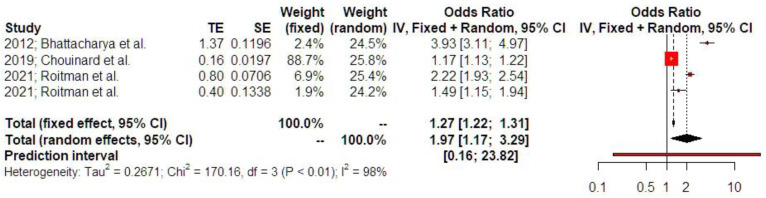
Odds ratio of emergency cesarean section. The vertical line represents the “no difference” point between the two groups. Red squares represent the odds ratios of the included studies; horizontal black lines indicate the 95% confidence intervals of the included studies; diamond represents pooled odds ratios obtained from the meta-analysis outcomes, together with a 95% confidence interval for all studies; the horizontal red line represents prediction intervals. The weight of the included research is illustrated separately for fixed and random effects models. High statistical heterogeneity is noted for emergency cesarean section (I^2^ = 98%) [1,12,16].

A history of ectopic surgery was positively associated with an increased risk of a subsequent emergency cesarean section (OR 1.97 [1.17; 3.29], findings from three studies). Prediction intervals indicated that the sample size that was used did not suffice to support these findings in future studies (Figure 8). P-curve analysis indicated the presence of evidential value and absence of p-hacking (Supplementary File S1).

## 4. Discussion

### 4.1. Principal Findings

The present systematic review and meta-analysis indicate a correlation between ectopic pregnancy and the occurrence of adverse perinatal outcomes in subsequent pregnancies. Specifically, they show a positive correlation between ectopic pregnancy and the occurrence of placental abruption, hypertensive disorders of pregnancy, and preterm birth in subsequent pregnancies. Additionally, they indicate that ectopic pregnancy is associated with the recurrence of ectopic pregnancy, emergency cesarean section, and the birth of a low-birth-weight newborn in the case of subsequent pregnancies.

In the analysis of the association between ectopic pregnancy and the occurrence of hypertensive disease of pregnancy, recurrence of ectopic pregnancy, preterm birth, and the performance of emergency cesarean section in subsequent pregnancies, statistical heterogeneity was observed, which could be attributed to the effects of small studies, something that was expected due to the relatively small number of participants included in the retrieved studies.

Although the prediction intervals indicated that the sample size used was insufficient to support these findings in future studies, the results of the P-curve analysis regarding the association with recurrent ectopic pregnancy, preterm birth, and emergency cesarean section showed that the likelihood of data manipulation (P-hacking) was absent, making our results indisputable.

### 4.2. Comparison to Existing Literature

#### 4.2.1. Ectopic Pregnancy and Emergency Cesarean Section

Regarding the high rates of cesarean sections in subsequent pregnancies, Bhattacharya et al. reported increased rates of cesarean sections, but did not distinguish between scheduled and emergency cesarean cases [12]. Similarly, Roitman et al. identified higher rates of cesarean sections in women with a history of ectopic pregnancy, attributing them to obstetric complications such as abnormal placentation or fetal distress [16]. Hoyos et al. and Tang et al. also reported increased rates of cesarean section, particularly after wedge resection for interstitial pregnancies or pregnancies in a cesarean scar [15,17]. The increased rates in these cases are justified by the higher likelihood of uterine rupture in the event of vaginal delivery.

#### 4.2.2. Ectopic Pregnancy and Recurrence of Ectopic Pregnancy

The increased risk of recurrent ectopic pregnancy (OR 1.97 [1.17; 3.29]) demonstrated in the results of our study is a well-established conclusion in the international literature. Lund Kårhus et al. demonstrated that women with a history of a first ectopic pregnancy face a higher risk of future ectopic pregnancies compared to women with a history of a first intrauterine pregnancy [4]. Bhattacharya et al. noted that the recurrence rates of ectopic pregnancies mainly increase after surgical interventions for a previous ectopic pregnancy [12]. Sagiv et al. and Demirdag et al. discovered that salpingectomy and methotrexate treatment led to recurrence of ectopic pregnancy in subsequent pregnancies, but surgical management resulted in a slightly higher risk of recurrence [13,19]. Svirsky et al. demonstrated that early conception after methotrexate administration carries a risk of recurrence, but their study had limited generalizability due to the small sample size [11]. Our observation of an increased risk of recurrent ectopic pregnancy aligns perfectly with established medical knowledge and supports the requirement for early ultrasound screening for all subsequent pregnancies in women with a history of ectopic pregnancy.

#### 4.2.3. Ectopic Pregnancy and Low Birth Weight

Bhattacharya et al. found that subsequent pregnancies after an ectopic pregnancy had a higher risk of giving birth to low-birth-weight infants [12]. Roitman et al. demonstrated the existence of an association between previous ectopic pregnancy and low birth weight, as well as the birth of small for gestational age (SGA) infants [16]. Chouinard et al. observed similar results and proposed placental dysfunction as a possible etiological mechanism, possibly due to previous uterine interventions or altered endometrial receptivity [1]. Yousefnezhad et al. compared the medical and surgical treatments of tubal ectopic pregnancies and found that both groups exhibited higher rates of fetal growth restriction [20]. The alignment of our results with these findings suggests that a history of ectopic pregnancy may lead to intrauterine environmental changes that affect fetal development.

#### 4.2.4. Ectopic Pregnancy and Preterm Labor

The observed increased risk of preterm birth (OR 1.58 [1.31; 1.91]) is one of the most frequently reported adverse outcomes in the literature. Bhattacharya et al. found that the rates of preterm birth were significantly higher after an ectopic pregnancy compared not only to women whose first pregnancy resulted in a live newborn but also to women with a history of miscarriage [12]. Roitman et al. confirmed the findings, noting preterm birth as the main adverse perinatal outcome [16]. Chouinard et al. also identified a strong positive correlation between preterm birth and a history of ectopic pregnancy [1]. Demirdag et al. and Lund Kårhus et al. both noted increased rates of preterm birth regardless of the initial management method (either surgical or medical), suggesting a potential common pathophysiological mechanism related to uterine injury or systemic inflammation [4,13].

#### 4.2.5. Ectopic Pregnancy and Pregnancy-Induced Hypertensive Disorders

Chouinard et al. also observed a significant risk of gestational hypertension and preeclampsia in women with a previous ectopic pregnancy, suggesting that scarring or inflammation of the uterus may contribute to pathological placentation [1]. Roitman et al. similarly reported a moderate but significant increase in hypertensive disorders, further supporting our data [16]. Hoyos et al. and Tang et al., who focused on pregnancies after interstitial gestation and gestation on the scar of a cesarean section, also found higher rates of preeclampsia and gestational hypertension, possibly due to alterations in the anatomy of the uterus from previous surgical interventions [15,17]. These findings have significant clinical implications as they highlight the necessity of monitoring blood pressure during prenatal care for pregnant women with a history of ectopic pregnancy.

#### 4.2.6. Ectopic Pregnancy and Placental Abruption

Chouinard et al. found that women, regardless of age, who had experienced an ectopic pregnancy before achieving an intrauterine pregnancy had a 21% higher risk of placental abruption (RR 1.21 [1.04; 1.41]). Additionally, this study showed that women over the age of 30 with a history of ectopic pregnancy faced a 42% higher risk of placental abruption (RR 1.42 [1.16; 1.69]) [1]. The same authors also reported an increased risk of placenta previa in pregnancies following an ectopic pregnancy. Roitman et al. included 243,682 women in their study, of whom 1424 had a history of ectopic pregnancy, and found that 2.1% of women with a history of ectopic pregnancy experienced placental abruption in a subsequent pregnancy, compared to 0.6% of women without a history of ectopic pregnancy (*p* = 0.042) [16].

### 4.3. Clinical Application

According to the results of the meta-analysis, ectopic pregnancy appears to have a positive correlation with clinical entities, the presence of which renders a pregnancy high-risk, while leading to complications of gestation that increase the likelihood of maternal and neonatal morbidity and mortality. These complications are more frequent in non-specialized centers that do not have a pregnancy pathology department, due to the lack of interdisciplinary care.

The development of timely diagnostic tools and predictive models based on the mother’s history is of essential importance. These models could lead to the development of predictive algorithms that would be applied to women with a history of ectopic pregnancy, with the aim of prediction, early diagnosis, and the avoidance of adverse perinatal outcomes.

The quantification of adverse perinatal outcomes resulting from our research and the alignment of our findings with the international literature underscore the clinical significance of previous ectopic pregnancies and highlight the need for increased and systematic monitoring as well as personalized care in subsequent pregnancies.

### 4.4. Strengths and Limitations

Our meta-analysis was based on a systematic review of the international literature, with a thorough search that included several databases designed to minimize potential losses by eliminating temporal and linguistic restrictions. Our findings emerged from the analysis of data from 2,162,623 women, of whom 23,790 experienced adverse perinatal outcomes in subsequent pregnancies, and there was clear agreement among the included studies regarding the definition of the outcome under investigation. The present study is the first meta-analysis to examine potential associations between ectopic pregnancy and the occurrence of adverse perinatal outcomes in subsequent pregnancies, including all relevant data published to date.

We acknowledge that the present meta-analysis has several limitations. Several parameters may contribute to this. For example, the methodological and clinical heterogeneity observed in the included studies may lead to significant selection bias that could hinder the extraction of clear conclusions. Specifically, nine retrospective cohort studies were included in the present systematic review, with one of them being historically controlled and three of them based on registered data. Additionally, a series of case reports, a cross-sectional study, and a population-based longitudinal cohort were included. Therefore, the designs of the studies differ from one another, and while the results provided are significant, further studies should be designed to draw specific conclusions.

## 5. Conclusions

The results of our meta-analysis indicate that ectopic pregnancy is associated with the recurrence of ectopic pregnancy, preterm birth, and the performance of emergency cesarean section in subsequent pregnancies, as the provided information can be considered credible due to the absence of potential factors that might interfere or negatively influence the analyses of primary studies. It also indicates the association of ectopic pregnancy with the onset of gestational hypertensive disease, placental abruption, and the birth of a low-birth-weight newborn in subsequent pregnancies. Although the findings of the present study are encouraging, they should be considered preliminary and serve as a basis for future research as the retrieved data are scarce and cannot be deemed sufficient. Therefore, further research is needed that should take into account this information during methodological planning to ensure the introduction of all reported outcomes.

## Supplementary Materials

The following supporting information can be downloaded at: https://www.mdpi.com/article/10.3390/jcm14124112/s1, Supplementary File S1: Statistical analysis.

## Abbreviations

The following abbreviations are used in this manuscript:

EP	Ectopic pregnancy
OR	Odds ratio
CI	Confidence interval
PI	Prediction interval
CS	Cesarean section
REM	Random effects model
NOS	Newcastle–Ottawa scale
TAS	Trial sequential analysis
HELLP	Hemolysis, elevated liver enzymes, and low platelets
