# Supplementary material for: Impact of Ectopic Pregnancy on the Outcomes of the Subsequent Pregnancy: A Systematic Review and Meta-Analysis
Source: J Clin Med. 2025 Jun 10;14(12):4112. doi: 10.3390/jcm14124112 (PMC12194004; doi:10.3390/jcm14124112)

Second ectopic

```
Trim-fill analysis
                                       OR             95%-CI %W(random)
2012; Bhattacharya et al.         13.0000 [10.7971; 15.6524]       20.3
2012; Lund Karhus et al.           9.2000 [ 6.9688; 12.1455]       19.1
2019; Chouinard et al.            16.3300 [15.4267; 17.2862]       21.2
Filled: 2012; Bhattacharya et al. 20.5130 [17.0369; 24.6983]       20.3
Filled: 2012; Lund Karhus et al.  28.9858 [21.9561; 38.2660]       19.1

Number of studies combined: k = 5 (with 2 added studies)

                          OR             95%-CI     z  p-value
Random effects model 16.3300 [11.2410; 23.7230] 14.66 < 0.0001

Quantifying heterogeneity:
 tau^2 = 0.1700 [0.0501; 1.5555]; tau = 0.4123 [0.2238; 1.2472]
 I^2 = 91.0% [81.9%; 95.5%]; H = 3.33 [2.35; 4.72]

Test of heterogeneity:
     Q d.f.  p-value
 44.38    4 < 0.0001


Small-study effects

                                 OR             95%-CI         OR
95%-CI
2012; Bhattacharya et al.   13.0000 [10.7971; 15.6524]    17.1494 [14.2433; 2
0.6484]
2012; Lund Karhus et al.     9.2000 [ 6.9688; 12.1455]    16.7908 [12.7187; 2
2.1666]
2019; Chouinard et al.      16.3300 [15.4267; 17.2862]    16.7751 [15.8471; 1
7.7573]

Result of limit meta-analysis:

 Random effects model      OR             95%-CI     z     pval
    Adjusted estimate 16.9042 [15.4031; 18.5515] 59.60        0
  Unadjusted estimate 12.7916 [ 9.3150; 17.5659] 15.75 < 0.0001

Quantifying heterogeneity:
tau^2 = 0.0694; I^2 = 90.0% [73.3%; 96.3%]; G^2 = 0.0%

Test of heterogeneity:
     Q d.f.  p-value
 20.03    2 < 0.0001

Test of small-study effects:
  Q-Q' d.f.  p-value
 19.32    1 < 0.0001

Test of residual heterogeneity beyond small-study effects:
    Q' d.f.  p-value
  0.71    1   0.3990
```

```
P-curve

Results
 -----------------------
                    pBinomial   zFull pFull   zHalf pHalf
Right-skewness test     0.125 -13.433     0 -13.280     0
Flatness test           1.000  14.074     1  14.074     1
Note: p-values of 0 or 1 correspond to p<0.001 and p>0.999, respectively.
Power Estimate: 8% (99%-99%)

Evidential value
 -----------------------
- Evidential value present: yes
- Evidential value absent/inadequate: no
```

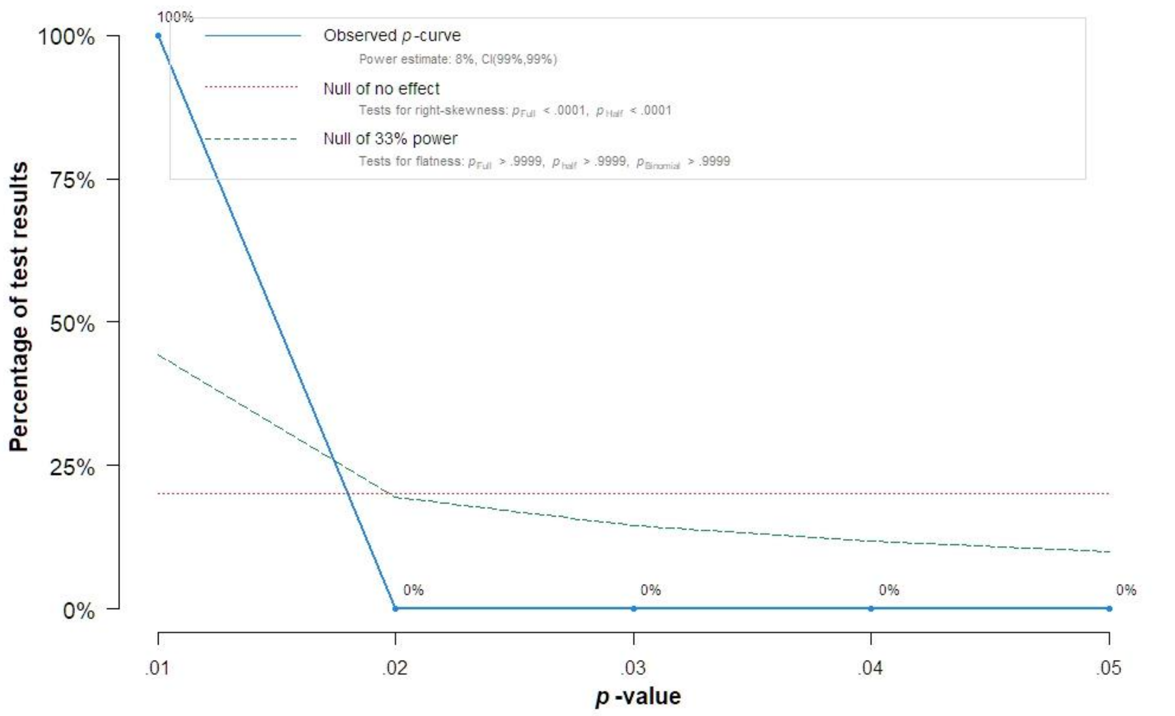

Note: The observed *p*-curve includes 3 statistically significant ($p < .05$) results, of which 3 are $p < .025$.
There were no non-significant results entered.

Placental abruption

Trim-fill analysis

```
                             OR           95%-CI %W(random)
2012; Bhattacharya et al.    1.3600 [0.8473; 2.1830]       19.8
2019; Chouinard et al.       1.2100 [1.0392; 1.4089]       31.9
2021; Roitman et al.         1.8815 [1.0362; 3.4162]       15.9
2021; Roitman et al.         1.9862 [0.7403; 5.3286]        8.2
Filled: 2021; Roitman et al. 0.7954 [0.4381; 1.4442]       15.9
Filled: 2021; Roitman et al. 0.7535 [0.2808; 2.0214]        8.2

Number of studies combined: k = 6 (with 2 added studies)

                         OR           95%-CI    z p-value
Random effects model 1.2450 [0.8999; 1.7223] 1.32  0.1858

Quantifying heterogeneity:
 tau^2 = 0.0799 [0.0000; 0.8481]; tau = 0.2827 [0.0000; 0.9209]
 I^2 = 17.6% [0.0%; 62.4%]; H = 1.10 [1.00; 1.63]

Test of heterogeneity:
    Q d.f. p-value
 6.07    5  0.2997
```

Small-study effects analysis

```
                          OR            95%-CI         OR            95%-CI
2012; Bhattacharya et al.   1.3600 [0.8473; 2.1830]    1.1288 [0.7032; 1.8118
]
2019; Chouinard et al.      1.2100 [1.0392; 1.4089]    1.1727 [1.0071; 1.3654
]
2021; Roitman et al.        1.8815 [1.0362; 3.4162]    1.2581 [0.6929; 2.2843
]
2021; Roitman et al.        1.9862 [0.7403; 5.3286]    1.1435 [0.4262; 3.0677
]

Result of limit meta-analysis:

 Random effects model     OR            95%-CI    z   pval
    Adjusted estimate 1.1747 [0.9347; 1.4764] 1.38 0.1674
  Unadjusted estimate 1.3603 [1.0579; 1.7490] 2.40 0.0164

Quantifying heterogeneity:
tau^2 = 0.0218; I^2 = 0.0% [0.0%; 84.7%]; G^2 = 4.2%

Test of heterogeneity:
    Q d.f. p-value
 2.92    3  0.4038

Test of small-study effects:
 Q-Q' d.f. p-value
 2.52    1  0.1125

Test of residual heterogeneity beyond small-study effects:
   Q' d.f. p-value
 0.40    2  0.8172
```

Emergency Cesarean section

Trim-fill analysis

```
        OR           95%-CI %W(random)
2012; Bhattacharya et al.        3.9300 [3.1088; 4.9681]       16.6
2019; Chouinard et al.           1.1700 [1.1256; 1.2161]       16.9
2021; Roitman et al.             2.2161 [1.9297; 2.5449]       16.8
2021; Roitman et al.             1.4947 [1.1499; 1.9430]       16.5
Filled: 2021; Roitman et al.     0.6242 [0.5435; 0.7168]       16.8
Filled: 2012; Bhattacharya et al. 0.3520 [0.2784; 0.4449]       16.6

Number of studies combined: k = 6 (with 2 added studies)

                         OR            95%-CI    z p-value
Random effects model 1.2224 [0.6121; 2.4414] 0.57  0.5693

Quantifying heterogeneity:
 tau^2 = 0.7379 [0.2809; 4.5136]; tau = 0.8590 [0.5300; 2.1245]
 I^2 = 98.6% [98.1%; 99.0%]; H = 8.58 [7.20; 10.21]

Test of heterogeneity:
      Q d.f.  p-value
 367.90    5 < 0.0001


   OR           95%-CI        OR            95%-CI
2012; Bhattacharya et al.   3.9300 [3.1088; 4.9681]    2.6152 [2.0687; 3.3059
]
2019; Chouinard et al.      1.1700 [1.1256; 1.2161]    1.1576 [1.1138; 1.2033
]
2021; Roitman et al.        2.2161 [1.9297; 2.5449]    1.9260 [1.6771; 2.2118
]
2021; Roitman et al.        1.4947 [1.1499; 1.9430]    0.9302 [0.7156; 1.2091
]

Result of limit meta-analysis:

 Random effects model     OR            95%-CI     z     pval
    Adjusted estimate 1.5261 [1.4444; 1.6124] 15.06 < 0.0001
  Unadjusted estimate 1.9659 [1.1745; 3.2906]  2.57   0.0101

Quantifying heterogeneity:
tau^2 = 0.2671; I^2 = 98.2% [97.1%; 98.9%]; G^2 = 91.0%

Test of heterogeneity:
      Q d.f.  p-value
 170.16    3        0

Test of small-study effects:
   Q-Q' d.f.  p-value
 123.33    1        0

Test of residual heterogeneity beyond small-study effects:
     Q' d.f.  p-value
  46.83    2 < 0.0001
```

```
P-curve analysis
 -----------------------
- Total number of provided studies: k = 4
- Total number of p<0.05 studies included into the analysis: k = 4 (100%)
- Total number of studies with p<0.025: k = 4 (100%)

Results
 -----------------------
                    pBinomial   zFull pFull   zHalf pHalf
Right-skewness test     0.062 -12.309     0 -11.990     0
Flatness test           1.000  11.661     1  11.963     1
Note: p-values of 0 or 1 correspond to p<0.001 and p>0.999, respectively.
Power Estimate: 99% (99%-99%)

Evidential value
 -----------------------
- Evidential value present: yes
- Evidential value absent/inadequate: no
```

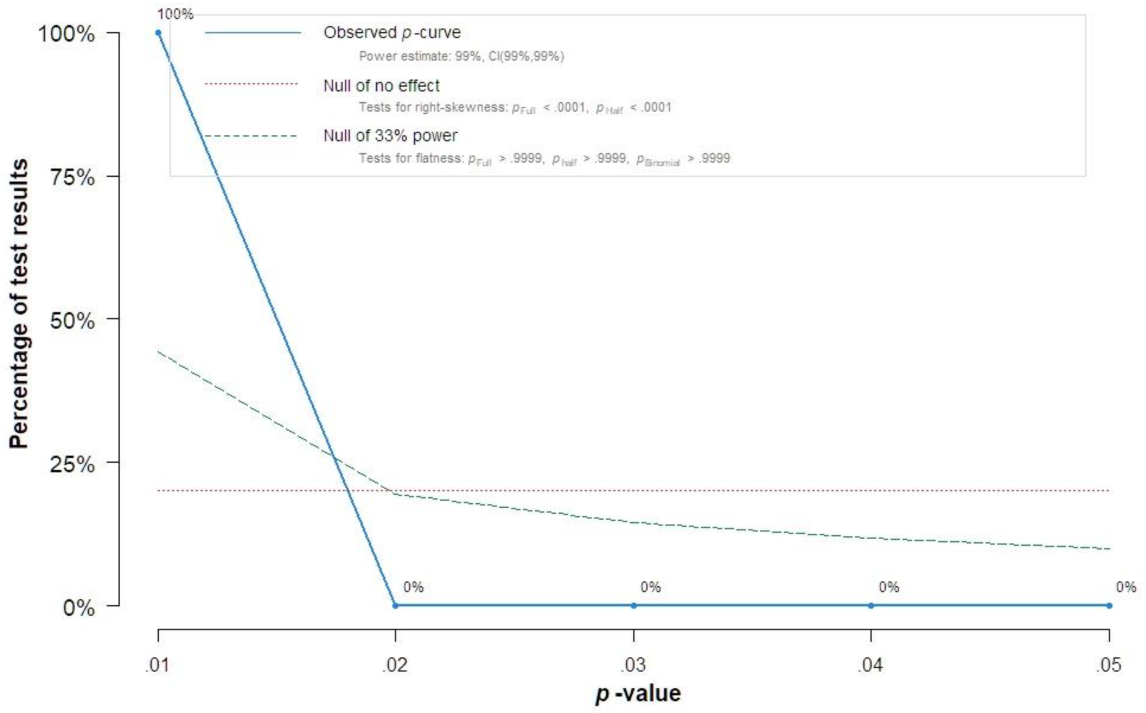

Note: The observed *p*-curve includes 4 statistically significant (p < .05) results, of which 4 are p < .025.
There were no non-significant results entered.

Hypertensive disorders of pregnancy

Trim-fill analysis

```
            OR             95%-CI %W(random)
2012; Bhattacharya et al.        2.1900 [1.4391; 3.3327]       15.2
2019; Chouinard et al.           1.1800 [1.0615; 1.3118]       20.3
2021; Roitman et al.             1.2378 [0.9625; 1.5919]       18.3
2021; Roitman et al.             1.4518 [0.9739; 2.1642]       15.6
Filled: 2021; Roitman et al.     0.9730 [0.6527; 1.4504]       15.6
Filled: 2012; Bhattacharya et al. 0.6450 [0.4239; 0.9816]       15.2

Number of studies combined: k = 6 (with 2 added studies)

                         OR            95%-CI    z p-value
Random effects model 1.1957 [0.8724; 1.6387] 1.11  0.2665

Quantifying heterogeneity:
 tau^2 = 0.1247 [0.0192; 0.9516]; tau = 0.3532 [0.1387; 0.9755]
 I^2 = 72.7% [37.2%; 88.1%]; H = 1.91 [1.26; 2.90]

Test of heterogeneity:
     Q d.f. p-value
 18.33    5  0.0026
```

Small-study effects analysis

```
OR            95%-CI        OR            95%-CI
2012; Bhattacharya et al.   2.1900 [1.4391; 3.3327]    1.2541 [0.8241; 1.9084
]
2019; Chouinard et al.      1.1800 [1.0615; 1.3118]    1.1351 [1.0211; 1.2619
]
2021; Roitman et al.        1.2378 [0.9625; 1.5919]    1.0228 [0.7953; 1.3154
]
2021; Roitman et al.        1.4518 [0.9739; 2.1642]    0.9567 [0.6418; 1.4261
]

Result of limit meta-analysis:

 Random effects model     OR           95%-CI    z   pval
    Adjusted estimate 1.0864 [0.9132; 1.2924] 0.94 0.3496
  Unadjusted estimate 1.4005 [1.0738; 1.8265] 2.49 0.0130

Quantifying heterogeneity:
tau^2 = 0.0511; I^2 = 64.7% [0.0%; 88.0%]; G^2 = 30.6%

Test of heterogeneity:
    Q d.f. p-value
 8.49    3  0.0369

Test of small-study effects:
 Q-Q' d.f. p-value
 5.82    1  0.0158

Test of residual heterogeneity beyond small-study effects:
   Q' d.f. p-value
 2.67    2  0.2633
```

Low birthweight

Trim-fill analysis

```
  OR            95%-CI %W(random)
2012; Bhattacharya et al. 1.2000 [0.7721; 1.8650]        9.2
2019; Chouinard et al.    1.2000 [1.0996; 1.3095]       51.2
2021; Roitman et al.      1.1957 [0.9563; 1.4951]       25.2
2021; Roitman et al.      1.6128 [1.1522; 2.2576]       14.4

Number of studies combined: k = 4 (with 0 added studies)

                         OR            95%-CI    z p-value
Random effects model 1.2509 [1.0820; 1.4462] 3.02  0.0025

Quantifying heterogeneity:
 tau^2 = 0.0087 [0.0000; 0.2792]; tau = 0.0934 [0.0000; 0.5284]
 I^2 = 0.0% [0.0%; 84.7%]; H = 1.00 [1.00; 2.56]

Test of heterogeneity:
    Q d.f. p-value
 2.82    3  0.4203
```

Small-study effects

```
   OR            95%-CI       OR            95%-CI
2012; Bhattacharya et al.   1.2000 [0.7721; 1.8650]    1.1202 [0.7208; 1.7411
]
2019; Chouinard et al.      1.2000 [1.0996; 1.3095]    1.1870 [1.0877; 1.2954
]
2021; Roitman et al.        1.1957 [0.9563; 1.4951]    1.1493 [0.9192; 1.4371
]
2021; Roitman et al.        1.6128 [1.1522; 2.2576]    1.3039 [0.9315; 1.8252
]

Result of limit meta-analysis:

 Random effects model     OR            95%-CI    z   pval
    Adjusted estimate 1.1881 [1.0357; 1.3629] 2.46 0.0138
  Unadjusted estimate 1.2509 [1.0820; 1.4462] 3.02 0.0025

Quantifying heterogeneity:
tau^2 = 0.0087; I^2 = 0.0% [0.0%; 84.7%]; G^2 = 7.1%

Test of heterogeneity:
    Q d.f. p-value
 2.82    3  0.4203

Test of small-study effects:
 Q-Q' d.f. p-value
 0.86    1  0.3533

Test of residual heterogeneity beyond small-study effects:
   Q' d.f. p-value
 1.96    2  0.3757
```

Preterm birth

Trim-fill analysis

```
OR            95%-CI %W(random)
2012; Bhattacharya et al.        1.8400 [1.3418; 2.5233]       15.4
2019; Chouinard et al.           1.2700 [1.1787; 1.3684]       20.1
2021; Roitman et al.             1.7146 [1.4160; 2.0762]       18.2
2021; Roitman et al.             1.8565 [1.3542; 2.5451]       15.4
Filled: 2012; Bhattacharya et al. 0.9489 [0.6920; 1.3013]       15.4
Filled: 2021; Roitman et al.      0.9405 [0.6860; 1.2893]       15.4

Number of studies combined: k = 6 (with 2 added studies)

                         OR            95%-CI    z p-value
Random effects model 1.3747 [1.0700; 1.7663] 2.49  0.0128

Quantifying heterogeneity:
 tau^2 = 0.0801 [0.0169; 0.5917]; tau = 0.2830 [0.1302; 0.7692]
 I^2 = 80.5% [57.8%; 91.0%]; H = 2.26 [1.54; 3.32]

Test of heterogeneity:
     Q d.f. p-value
 25.58    5  0.0001
```

Small-study effects

```
         OR            95%-CI         OR            95%-CI
2012; Bhattacharya et al.   1.8400 [1.3418; 2.5233]    1.2763 [0.9307; 1.7503
]
2019; Chouinard et al.      1.2700 [1.1787; 1.3684]    1.2403 [1.1511; 1.3364
]
2021; Roitman et al.        1.7146 [1.4160; 2.0762]    1.4435 [1.1921; 1.7479
]
2021; Roitman et al.        1.8565 [1.3542; 2.5451]    1.2849 [0.9372; 1.7614
]

Result of limit meta-analysis:

 Random effects model     OR            95%-CI    z     pval
    Adjusted estimate 1.3090 [1.1626; 1.4738] 4.45 < 0.0001
  Unadjusted estimate 1.5829 [1.3105; 1.9120] 4.77 < 0.0001

Quantifying heterogeneity:
tau^2 = 0.0245; I^2 = 81.4% [51.5%; 92.9%]; G^2 = 12.1%

Test of heterogeneity:
     Q d.f. p-value
 16.12    3  0.0011

Test of small-study effects:
  Q-Q' d.f. p-value
 15.04    1  0.0001

Test of residual heterogeneity beyond small-study effects:
    Q' d.f. p-value
  1.08    2  0.5814
```

```
P-curve analysis
 -----------------------
- Total number of provided studies: k = 4
- Total number of p<0.05 studies included into the analysis: k = 4 (100%)
- Total number of studies with p<0.025: k = 4 (100%)

Results
 -----------------------
                    pBinomial  zFull pFull  zHalf pHalf
Right-skewness test     0.062 -8.033     0 -7.669     0
Flatness test           1.000  5.956     1  6.451     1
Note: p-values of 0 or 1 correspond to p<0.001 and p>0.999, respectively.
Power Estimate: 99% (98%-99%)

Evidential value
 -----------------------
- Evidential value present: yes
- Evidential value absent/inadequate: no
```

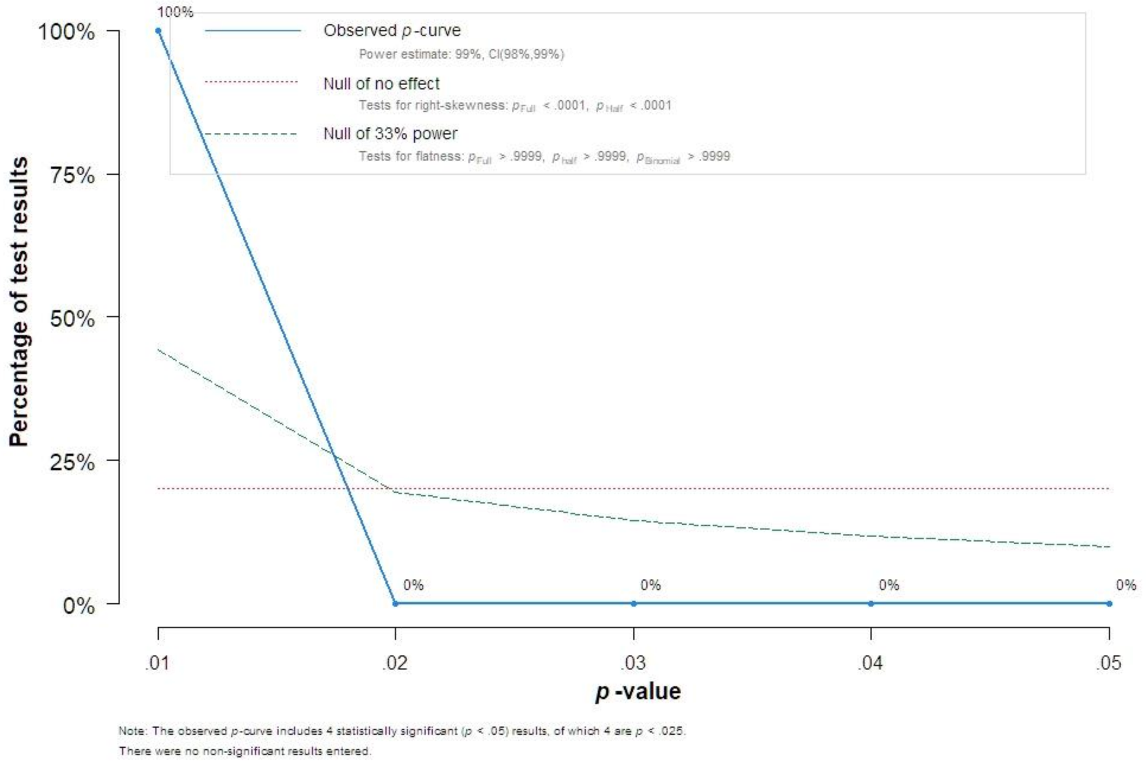

Supplement: Supplementary file 1 [file jcm-14-04112-s001.zip › jcm-3640009-supplementary.pdf]
